# 

**Published:** 1880-10

**Authors:** 


### THE BISTOURY :

Thad S. Up de Graff, M. D., Editor & Proprietor.

### *CONTENTS FOR OCTOBER, 1880.*

**CONTRIBUTIONS.** PAGE.

The Universality of Lying.........................BEN. H. PRATT,.............................. 282

The Law's Charlatanry,,..........................AUSBURN TOWNER.............................. 284

A Smile,.........................................J. J. KEYES.................................. 287

Crystals for the Microscope,.....................GEO. E. BLACKHAM, M. D., F. R. M. S............... 289

"Penology,"......................................J. B. CHANDLER............................... 290

**MEDICAL ITEMS AND NEWS.**

Chronic Eczema—Coca Cure for Opium-Habit—Rheumatism—Burns—Vomiting of Pregnancy—Copaiba-Mixture—Diphtheria—Antidote for Strychnia—Oleate of Lead—Tetanus—Mistura Balsami Copaivæ—Signs of Death—Vaginitis—Duboisia and Atropia—Internal Use of Tar—Caustic Crayons—Chloroform Gargle—Administration of Castor Oil—Chian Turpentine—Diabetes—Chronic Laryngitis—Cedron as Substitute for Quinine—A New Anthelmintic—Gonorrhœa—Chronic Eczema—Oil of Tansy—Relation of Diarrhœa to Temperature—Citrate of Caffeine—Sick Headache—Administering Kooso—Local Anæsthesia with Bromide of Ethyl—Dyspnœa—To Facilitate the Exhibition of Iron—Chest Diseases—Fits of Sneezing—Hay Fever—Prurigo—Nitrate of Amyl—Ulcers of the Cervix—Aspidium Marginale in Tapeworm—Bladder and Kidney Ailments—The Opium Habit—Diabetis Insipidus—Catarrh-Skin Diseases—Therapeutic Value of Pulsatilla—Asthma—Treatment of Constitutional Syphilis by Sulphate of Copper—Cough—Tonga—Some of the Formulas of the Hartford, Conn, Hospital—Medicated Pencils.........................................292 301

**HOUSEHOLD HINTS AND RECIPES.**

Ink Stains—Rancid Butter—Mouth Wash—Gold Varnish for Metals—Chicken Cholera—Bread as a Digestive Agent—Waterproofing Boots—To Age Oak Furniture—Clothes Wringer as Press—Improved Polishing Cloth—Colored Inks—To Render Fabric Incombustible—To Prevent Glue from Cracking—Liquid Glue—Marking-Ink for Linen, etc—Cement for Rubber—Cleaning Paint Brushes—Artificial Vanillen from Oil of Cloves—Black Bricks.........................................302 to 304

**OUR CORRESPONDENCE** One Letter.........................................304

**EDITORIAL,**

THE NEW MEDICAL LAW.........................................305
INDICATIONS.........................................306

**CHIT-CHAT.**

Harassing—Desirable Premium—That Home—Novel Idea—Rev. E. P. Adams' Case—Those Two Societies—An Invention—Order Asked For—The Elmira Microscopical Society—Locomotive Whistles—Miss Neilson—Cardinal Flower—The Only Way—A Companion Dies.........................................307 to 309

